# Imatinib in Chronic Myeloid Leukemia: an Overview

**DOI:** 10.4084/MJHID.2014.007

**Published:** 2014-01-02

**Authors:** Tomasz Sacha

**Affiliations:** Department of Hematology, Collegium Medicum, Jagiellonian University, Cracow, Poland.

## Abstract

Imatinib was the first signal transduction inhibitor (STI), used in a clinical setting. It prevents a BCR-ABL protein from exerting its role in the oncogenic pathway in chronic myeloid leukemia (CML). Imatinib directly inhibits the constitutive tyrosine kinase activity. Imatinib binds to BCR-ABL kinase domain by preventing the transfer of a phosphate group to tyrosine on the protein substrate and the subsequent activation of phosphorylated protein. As the result, the transmission of proliferative signals to the nucleus is blocked and leukemic cell apoptosis is induced. The FDA has approved imatinib as first-line treatment for newly diagnosed CML in December 2002 following an International Randomized Study (IRIS), initiated in June 2000, comparing imatinib at a single daily dose 400 mg to IFN alpha plus cytarabine in newly diagnosed patients with CML in CP. Results from this study show the outstanding effectiveness of imatinib and its superiority with respect to the rates of complete hematological response (CHR), major and complete cytogenetic response (MCyR, CCyR). Patients randomized to imatinib arm at 8 – year data cut off continue to have a durable hematologic and cytogenetic responses, low progression rates to AP or BC, and remarkable survival outcomes. An overall survival (OS) rate is 85% for patients receiving imatinib (93% when only CML-related deaths and those prior to stem cell transplantation are considered). The results have been confirmed in the last years by several groups. According these cumulative results the rates of CCyR achieved after one year of therapy with imatinib at standard dose ranged from 49% to 77%, and the proportion of patients who achieved major molecular response (MMR) after one year ranged between 18% and 58%. Discontinuation of imatinib has been also tried in patients in MMR, a molecular relapse occurs in about one third of patients, generally within 6 months from imatinib cessation.

## Introduction

Imatinib was the first signal transduction inhibitor (STI), used in a clinical setting. It prevents a BCR-ABL protein from exerting its role in the oncogenic pathway in chronic myeloid leukemia (CML). Imatinib directly inhibits the constitutive tyrosine kinase activity, which results in the modification of the function of various genes involved in the control of the cell cycle, cell adhesion, cytoskeleton organization and finally in the apoptotic death of Ph(+) cells.^1^

Imatinib binds to BCR-ABL kinase domain, which is in an inactive conformation in a pocket reserved for the ATP binding site, thus preventing the transfer of a phosphate group to tyrosine on the protein substrate and the subsequent activation of phosphorylated protein. As the result, the transmission of proliferative signals to the nucleus is blocked and leukemic cell apoptosis is induced.^2^ Preclinial in vitro studies showed that imatinib is a tyrosine kinase inhibitor (TKI) of ABL kinase and its active derivatives: viral Abelson nonreceptor protein tyrosine kinase (v-ABL), BCR-ABL,^3^,^4^ TEL-ABL,^5^ platelet-derived growth factor receptor (PDGFR) and Steel factor receptor (c-KIT) kinases.^6^ Imatinib exhibits high level of selectivity. Its activity against the above protein kinases is similar with IC50 values in the range of 0.025 M for protein autophosphorylation and is at least 100-fold lower than for a large number of other tyrosine and serine/threonine kinases.^6^

## Pharmacokinetics of Imatinib

Pharmacokinetics of imatinib is characterized by rapid and complete oral bioavailability (98%) and a proportional dose-exposure relationship.^18^,^19^ There is no significant interaction of imatinib with food intake. Its terminal half-life is approximately 18 hours, allowing for once-daily dosing.^18^,^19^ The median peak plasma concentrations at steady state of imatinib administered orally once a day at a dose of 400 mg and median trough levels are 5.4 M, and 1.43 M respectively.^7^ Imatinib is metabolized by the cytochrome P450 system. CYP3A4 is the major isoenzyme responsible for imatinib metabolism, although CYP1A2, CYP2D6, CYP2C9, and CYP2C19 also contribute to a minor extent.^18^,^19^ The activity of CYP enzyme exhibits intrinsic variability, which could be the cause of high interpatient unevenness in imatinib exposure.^18^,^19^ Drugs that are inhibitors or inductors of the CYP3A4 isoenzyme have been shown to alter imatinib pharmacokinetic activity.^20^

## Efficacy of Imatinib

### Phase I trials

The first phase I trial was initiated in June 1998 and enrolled patients suffering from CML in chronic phase (CP) who were resistant to or intolerant of interferon alpha (IFN alpha). Almost all patients (98%) treated with at least 300 mg imatinib per day achieved complete hematological response (CHR). Major and complete cytogenetic response (MCyR, CCyR) were obtained by 31% and 13% of patients respectively. Of note, the responses were durable, only 2 relapses (out of 53 patients) were noted after a median follow-up of 265 days.^8^ Based on these results, the protocol was expanded and included 58 patients in blast crisis (BC) or with Ph-positive acute lymphoblastic leukemia (Ph+ALL). Hematological responses to minimal dose of imatinib in this cohort (300 mg daily) were obtained in 55% and 70% of patients with myeloid, and lymphoid blast crisis respectively, including four CHR in each group. Twelve percent of patients achieved MCyR and 8% CCyR. Patients treated in BC unlike patients treated in the CP relapsed frequently after initiating imatinib therapy. Approximately 50% of responders with myeloid phenotype and all but one responder with lymphoid phenotype relapsed between 42 and 193 days of imatinib treatment (Druker et al., 2001b). Patients in accelerated phase (AP) had not been studied in the phase I protocols.

### Phase II trials

Three international multicenter phase II trials were initiated in 1999. The study population included patients with CML in myeloid BC, relapsed Ph+ALL, CML in AP, and patients who were resistant to IFN alpha. The results achieved in patients suffering from BC with myeloid phenotype largely confirmed the results obtained in the phase I study.^9^ In contrast to patients with myeloid type of disease, none of patients treated for lymphoid blast crisis and for relapsed Ph+ALL had durable response to imatinib.^10^ The results of patients treated for AP fall between those observed in myeloid BC and CP with the 1-year survival achieved in 74% patients. A retrospective comparison between two dose cohorts (400 mg and 600 mg daily) showed a significantly longer time to progression and overall survival for the 600 mg cohort.^11^ Based on these studies the recommended daily dose for patients in advanced phases of CML was set at 600 mg daily,^9^,^11^ and the recommendation of increasing the imatinib dose to 600 and 800 mg daily was rapidly extended also to patients in CP with unsatisfactory response to 400 mg daily, or response loss. The patients suffering from CML in CP hematologically or cytogenetically resistant or refractory, or intolerant of IFN alpha were the largest group studied within phase II trials. Ninety five percent of all patients achieved CHR; CCyR and MCyR were seen in 41% and 60% of patients respectively, and the progression-free survival rate at 18 months was 89%.^6^

### Phase III trials

An International Randomized Study of Interferon and STI571 (IRIS) comparing imatinib at a single daily dose 400 mg to IFN alpha plus cytarabine in newly diagnosed patients with CML in CP was initiated in June 2000. Results from this study show the outstanding effectiveness of imatinib and its superiority with respect to the rates of CHR, MCyR and CCyR. At 18 months, the rate of CCyR in patients treated with imatinib was 76% versus 15% for patients treated with IFN alpha plus cytarabine (*P* < .001). Importantly, the rate of progression to AP or BC at 18 months wassignificantly reduced in patients randomized to imatinib arm.^12^ Based on these results, the FDA has approved imatinib as first-line treatment for newly diagnosed CML in December 2002. At 8 years of follow-up, 45% of patients had discontinued treatment due to adverse events (AEs)/safety (6%), unsatisfactory therapeutic outcome (16%), stem cell transplantation (SCT) (3%), death (3%) or other reasons (17% for withdrawal or lack of renewal of consent and miscellaneous). Patients randomized to imatinib arm at 8 – year data cut off continue to have a durable hematologic and cytogenetic responses, low progression rates to AP or BC, and remarkable survival outcomes.^12^–^15^ An overall survival (OS) rate is 85% for patients receiving imatinib (93% when only CML- related deaths and those prior to stem cell transplantation are considered), with the annual rates of progression to AP or BC in year 4 to 8 after imatinib therapy onset are: 0.9%, 0.5%, 0%, 0%, and 0.4%, respectively. Progression to AP or BC was noted only in 3% of patients who achieved CCyR, and in none of patients who achieved major molecular response (MMR, < 0.1% BCR-ABL/control gene ratio on international scale) at 12 months of therapy.^16^ The patients treated with imatinib who had at 18 months a ≥ 3 log reduction in the level of BCR-ABL transcripts compared with a standardized baseline had a minimal risk of disease progression over the subsequent 12 months.^17^

The achievement of an MMR continued to be associated with an improved outcome at 5-year, with estimated rates without progression to AP/BC of 100%, 98%, and 87% for patients achieving CCyR and MMR, CCyR without MMR, and no CCyR, respectively.^13^ The best observed MMR rate with the 8-year follow-up of IRIS trial is 86%. The results of imatinib first line based on analysis of data derived from clinical trials and registries have been reported in the last three years by several groups (PETHEMA, SPIRIT, GIMEMA, CAMELIA, German Study Group IV, and others.).^21^–^30^ The rates of CCyR achieved after one year of therapy with imatinib at standard dose ranged from 49% to 77%, and the proportion of patients who achieved MMR after one year ranged between 18% and 58%.

## The Efficacy of Imatinib at Higher Dose; Combinations with other Agents

Preclinical data and some observations from single – arm studies suggested that higher dosages of imatinib could be more effective than standard 400 mg once daily dose, and may provide a better disease control. The amplification of the BCR-ABL gene or overexpression of bcr-abl protein kinase are two known mechanisms of relative resistance to imatinib^34^–^36^ which could be overcome by dosages of 600 mg or 800 mg daily. However the initial reports that high dose imatinib treatment results in better, and achieved more rapidly responses than during standard dose imatinib remain controversial. Kantarjian et al. reports that compared with standard- dose imatinib, the dose of 800 mg daily was associated with achievement of significantly better rates of CCyR, major (defined as BCR-ABL/ABL ratio ≤ 0.05%), and complete (BCR-ABL/ABL negative) molecular responses. Transformation-free survival in this cohort of patients was significantly better with high-dose imatinib. Similar frequency of common adverse events with that seen with standard-dose imatinib were reported. The most common causes for dose reduction were myelosuppression.^37^ The German Study Group IV randomized trial compared imatinib 800 mg daily with standard dose imatinib +/− IFN in newly diagnosed CML patients in CP with regard to molecular response at 12 months and survival. Of 218 patients receiving imatinib 800 mg and evaluable for dosage at 12 months 45.9% received more than 700 mg/day. The cumulative incidences of CCyR and MMR at 12 months were significantly higher in imatinib 800 mg arm and lower and comparable in imatinib 400 mg and imatinib 400 +IFN alpha arms. MMR at 12 months have been reached faster with imatinib 800 mg, but this faster response did not translate into a better OS or progression-free survival (PFS).^25^ A Randomized European LekemiaNet Study addressed the issue of comparison of imatinib 400 mg and 800 mg daily in the front-line treatment of high-risk, Philadelphia- positive CML patients. 216 high risk patients according to the Sokal index were randomized into a group treated for at least 1 year front – line with imatinib at a dose of 800 mg daily or 400 mg daily. At 12 months the rate of CCyR (the primary endpoint) was better in the high-dose arm than in the standard-dose arm but the difference was not statistically significant. Importantly, the number of failures and the number of patients who discontinued treatment for any reasons were not different in those two arms. The proportion of MMR at any time point was slightly but nonsignificantly higher in the high-dose arm than in the standard-dose arm. This large, prospective, intention-to-treat, randomized study on therapy for high risk patients with CML failed to demonstrate a benefit of imatinib administered at a higher dose for the primary end point, as well as for any other measure of efficacy, toxicity, and compliance.^38^ In the other study of imatinib 400 mg versus 800 mg daily in 227 patients in late chronic phase who were resistant or intolerant to IFN alpha imatinib 800 mg daily was associated with a higher CCyR rate at 6 months but not at 12 months.^39^ The MMR rate at 12 months was the primary end point of another study of 476 patients, any risk, who were randomized to receive front-line imatinib at a dose of 800 mg or 400 mg daily.^40^ The difference in the MMR rate at 12 months between 400 mg and 800 mg arm was not significant. These studies does not support the wide use of imatinib at higher dosis front-line in all patients suffering from CML.

Combination of imatinib with low dose arabinosyl cytosine have been tested in two randomized trials, but none have demonstrated a superiority versus therapy with imatinib alone.^41^,^22^ The German CML Study IV was designed to compare in a randomized fashion standard imatinib vs. imatinib + IFN alpha vs. imatinib + low dose araC vs. imatinib after IFN (for low- and intermediate-risk patients) or vs. imatinib 800 mg (for high-risk patients). At 3 years, the cumulative incidence of CHR, MCyR, CCyR and of MMR were comparable for primary imatinib therapies.^41^ A French SPIRIT randomized study for untreated chronic-phase CML patients compared efficacy of imatinib alone at a dose of 400 mg daily, imatinib (400 mg daily) plus cytarabine (20 mg per square meter of body-surface area per day on days 15 through 28 of each 28-day cycle) or pegylated interferon (peginterferon) alfa-2a (90 μg weekly), or imatinib alone at a dose of 600 mg daily.^22^ At 12 months, the rates of CCyR were similar among the four groups. Combination of imatinib and low dose arabinosyl cytosine was not superior to imatinib alone in any measure of efficacy. Patients treated with combination of imatinib and peginterferon alfa-2a achieved significantly better rate (30%, 38%) of a superior molecular response (corresponding to MR 4) than patients receiving 400 mg of imatinib alone (14%, 21%) at 1, and at 2 year respectively (P = 0.001). During the first year of the trial, however, 39% of the patients discontinued cytarabine, and 45% discontinued peginterferon alfa-2a, both predominantly due to a toxicity. A lower dose of peginterferon alfa-2a (e.g., 45 μg per week) enhanced the tolerability while retaining the antileukemic efficacy. The rate of grade 3 to 4 hematologic toxicity has been reduced from 54% to 27%, and the proportion of patients who discontinued peginterferon alfa-2a before 6 months decreased from 40% to 10%. By 12 months, the cumulative MR4 for the subgroup treated with imatinib at a dose of 400 mg and the PegIFN90 vs imatinib of 400-mg and PegIFN45 were 25% and 28% respectively.^42^ The main phase of CML German Study IV compared monotherapy with imatinib 400 mg/d versus imatinib 400 mg/d combined with nonpegylated IFN-alpha given at an initial dose of 1.5 mill.U three times per week and increased up to 3 mill.U three times per week, according to tolerability versus imatinib 800 mg/d. At 12 months the rates of MMR which was the first primary end point were similar in the monotherapy imatinib 400 mg/d arm (44% [95% CI, 37% to 50%]) and in the imatinib 400 mg/d combined with IFN-alpha 46% [95% CI, 40% to 52%] arm and inferior to imatinib 800 mg/d arm.^25^ In the Nordic trial newly diagnosed chronic-phase CML patients with a low or intermediate Sokal risk score were randomized either to group treated with a combination of pegylated IFN-alpha 2b (Peg–IFN-alpha 2b) at a dose of 50 μg weekly and imatinib 400 mg daily or with imatinib 400 mg daily as a monotherapy. The MMR rate at 12 months was significantly higher in the combination arm (82%) compared with the imatinib monotherapy arm (54%; intention-to-treat, *P=* .002). In the combination arm, however, 61% of patients discontinued Peg–IFN-alpha 2b, most because of toxicity.^43^ In the MD Anderson Cancer Center (MDACC) trial patients were randomized to receive imatinib 400 mg twice daily, and combination of imatinib 800 mg/d with pegylated rIFN-alpha 2b 0.5 μg/kg weekly. The MMR and the CCyR rates were comparable in both arms.^44^ None of these combination studies has demonstrated a superior PFS or OS for patients who received combined treatment.

## Studies on Cessation of Imatinib

Allogeneic – haematopoietic stem cell transplantation (Allo-HSCT) has been and is still considered as the sole treatment able to cure CML. As a result of allo-HSCT a long period of time free from cytogenetic or hematologic relapse of the disease without the need for maintenance therapy could be achieved.^45^,^46^ However a thorough monitoring of minimal residual disease with qRT-PCR could demonstrate a presence of the BCR-ABL gene transcript even a long time after transplantation, which does not necessarily imply relapse, because no other signs of disease recurrence were observed.^47^,^48^ Apparently most of the patients were cured even though not all BCR-ABL positive leukemic cells were completely eradicated. A similar pattern is now observed in the course of the long-term follow-up of TKI-treated patients who eventually stop the treatment after achieving a deep and sustained molecular remission, and is a convincing illustration of the concept of operational cure”.^49^ The first pilot study was initiated in 12 chronic phase CML patients treated with imatinib at a standard dose who have achieved and maintained a complete molecular response (CMR) for at least 2 years, which was assessed by qRT-PCR with a sensitivity ranging between a 4.5- and a 5-log reduction. After a median follow-up time of 18 months, 50% of patients remained off-therapy without reappearance of BCR-ABL transcripts.^50^ Those patients still have an undetectable level of BCR-ABL transcripts after a median follow-up time of 6 years (range, 4–8). The same entry criteria were used for the multicenter study entitled the “Stop Imatinib” (STIM) which enrolled prospectively one hundred patients. The treatment with imatinib was restarted in the case of molecular relapse, which was arbitrarily defined as 2 positive qRT-PCR results over a period of 1 month showing at least a 1 log increase in BCR-ABL transcripts. At 36 months the overall probability of molecular relapse–free, and treatment-free remission was 39% (95% CI, 29–48). Most patient relapsed within 6 months from imatinib cessation; 3 cases of late relapse occuring at months 19, 20, and 22, respectively were noted. Most patient form the pilot as well as from the STIM study remained responsive to retreatment with imatinib. The second French “Stop Imatinib” (STIM2) study used the same criteria as those for the STIM1. The molecular relapse was defined also in a similar way, and a loss of MMR detected at one point was a trigger of TKI retreatment. The median follow-up of 124 enrolled patients is 12 months (range 1–25). After discontinuation of imatinib, a molecular relapse occurred in 48 pts (most within 6 months form imatinib cessation; 3 relapses between 6 and 12 month), and 76 patients (61%) were still free-of treatment at the last update. Forty one patients experienced a BCR-ABL transcript fluctuation in the qRT-PCR without clear molecular relapse.^52^ The TWISTER study is a prospective clinical trial which have used very similar as those in a STIM study entry and molecular relapse criteria for 40 chronic phase CML patients who discontinued imatinib. At 24 months, the proportion of patients remaining in stable treatment-free remission was 47.1%. Most patients relapsed within 4 months of stopping imatinib, importantly, no relapses beyond 27 months were observed.^53^ Other report demonstrates the probability of maintaining the CMR at 1 year of 28,6% after discontinuation of imatinib in 14 chronic phase CML patients. None of the patients however was strictly in CMR throughout the entire 2-year period preceding cessation of imatinib and half of the patients were high risk according to the Sokal index.^54^ A nationwide survey conducted in Japan identified 50 patients who stopped imatinib for at least 6 months. Molecular relapse was detected in 19 out of 43 analyzed patients, and the CMR rate after imatinib discontinuation was estimated to be 47%.^55^ All mentioned above studies demonstrate the proof of concept for stopping imatinib in CML patients who were able to achieve a deep, sustained molecular response. It seems certain that an MMR is not enough to plan a discontinuation strategy. The STIM1 and STIM2 study used a 4.5 – 5-log reduction, and the TWISTER study used a 4.5-log reduction for their definition of CMR. Larger studies suggest that beside of the level of BCR-ABL transcripts after imatinib treatment, the duration of deep molecular response is of major importance in achievement of a long-term treatment-free survival. In the STIM1, STIM2 and TWISTER trials, a sustained CMR for at least 2 years was used as the criterion.^51^,^52^,^53^ In the multivariate analysis and logistical regression in the STIM1 study, Sokal risk and imatinib therapy duration were confirmed as 2 independent prognostic factors for prediction of molecular relapse after imatinib discontinuation.^51^ It is obviously necessary to monitor regularly the minimal residual disease using qRT-PCR to allow an early detection of a fast molecular recurrence and restart the treatment as soon as possible. Most of the molecular recurrences occurred within the first few months of imatinib cessation. The presence of residual BCR-ABL positive cells in CML patients in CMR before and after imatinib discontinuation were demonstrated by Ross et al. who used genomic DNA-based PCR, which allows to detect rearranged BCR-ABL gene at a level of around 1- to 2-log below the detection limit of conventional (mRNA) qRT-PCR. However, there was no link between detection of BCR-ABL by genomic DNA-based PCR and relapse.^53^ Monitoring of residual disease by more sensitive conventional qRT-PCR within STIM1 study also does not allow the prediction of relapse after imatinib cessation.^51^ Importantly some fluctuations in BCR-ABL levels detected by conventional qRT-PCR method (in 33% patients in STIM2 study) could be observed after discontinuation without confirmation of a molecular relapse. 41 patients (33%) in STIM2 study experienced a BCR- ABL qRT-PCR fluctuation without molecular relapse, confirming that BCR-ABL reappearance does not mean automatically clinical relapse and reinforcing the concept of “operational cure”.^52^,^49^

## Figures and Tables

**Table:** Efficacy of imatinib in front line treatment in major clinical trials

Trial	Studied population	Imatinib dosage	Complete cytogenetic response (CCyR) rate [%]	Major molecular response (MMR) rate [%]	Estimated progression-free survival (PFS)/, overall survival (OS)

Phase I trials					

[Druker et al. 2001a]	CML patients in chronic phase (CP) resistant to or intolerant of IFN alpha	≥ 300 mg/d	13%	NR	NR

[Druker et al. 2001b]	CML patients in blastic phase – myeloid	300–1000 mg/d	14%	NR	PFS: 84 days/OS: NR
	CML patients in blastic phase – lymphoid		14%	NR	PFS: 58 days/OS: NR

Phase II trials					

[Sawyers et al. 2002]	CML patients in blastic phase – myeloid	400–600 mg/d	7%	NR	Median response time 10 months/median survival time 6,9 months

[Ottmann et al. 2002]	CML patients in blastic phase – lymphoid	400–600 mg/d	17%	NR	2,2/4,9 months

[Deininger et al. 2003]	CML patients in CP resistant or refractory, or intolerant of IFN alpha	400 mg/d	41%	NR	18 month PFS 89%/9,2 months

Phase III trials					

IRIS study [Deininger et al. 2009]	CML patients in CP de novo	400 mg/d	At 8 years: 83%	At 8 years: 86%	At 8 year: PFS:92%/OS: 89%; 93% if only CML – related deaths considered

PETHEMA [Cervantes et al. 2010]	CML patients in CP de novo	400 mg/d	At 3 years (ITT) 78,8%	At 3 years (ITT) 63%	At 5 years PFS: 94,3%/OS: 97,5%

SPIRIT [Preudhomme et al. 2010]	CML patients in CP de novo	400 mg/d	At 12 months 58%	At 12/24 months: 38%/43%	At 2 years PFS: 97,5%

GIMEMA [Gugliotta et al. 2011]	CML patients in CP de novo	400 mg/d	At 52 months 87%–88%	At 52 months 85%	At 6 years PFS:75/90%/S: 78/92% (<65/≥ 65 year-old pts.resp.)

CAMELIA [Faber et al. 2013]	CML patients in CP de novo	400 mg/d	83%	NR	At 5 years PFS:96%/OS: 90%

German Study Group IV [Hehlmann et al. 2011]	CML patients in CP de novo	400 mg/d	At 3 years 85,2%	At 3 years 79%	At 3 years PFS:94–99%/OS:93–99% (for <1% and ≥1% of BCR-ABL resp.)

DASISION [Saglio et al. 2010]	CML patients in CP de novo	400 mg/d	At 18 months 70%	At any time 41%	PFS: 93,7%/OS: 97,9%

ENESTnd [Kantarjian et al. 2010]	CML patients in CP de novo	400 mg/d	At 12 months 65%	At any time 44%	PFS 95,2%/OS: 96,4%

Combinations of IM and high IM dose					

[Kantarjian et al. 2004]	CML patients in CP de novo	800 mg/d	90%	63% (BCR-ABL/ABL ratio <0,05%)	At 15 months PFS:98%/OS: 98,3%%

European LeukemiaNet study [Baccarani et al. 2009]	CML patients in CP de novo	400 mg/d	At 12months 58%	At 12 months 33,3%	At 3 years PFS: 86%/OS: 84%
		800 mg/d	At 12 months 64%	At 12 months 39,8%	At 3 years PFS: 88%/OS: 91%

[Andreas et al. 2008]	CML patients in CP de novo	400 mg/d	At 6 months 20%	At 6 months 7%	NR
[Andreas et al. 2008]	CML patients in CP de novo	800 mg/d	44% (differences not significant at 12 months)	20% (differences not significant at 12 months)	NR

TOPS study [Cortes et al. 2008]	CML patients in CP de novo	400 mg/d	At 12 months 66%	At 12 months 40,1%	AT 18 months PFS: 95%/OS: 98,7%
		800 mg/d	66%	46,4%	PFS: 97%/OS: 98,2%

German Study Group IV [Hehlmann et al. 2011]	CML patients in CP de novo	800 mg/d	At 3 years 85,2%	At 3 years 79%	At 3 years PFS:94%/99%/OS: 93/99% (for <1% and ≥1% of BCR-ABL resp.)
		400 mg/d + IFN alpha	78,5%	63%	At 2 years PFS: 80,3%/at 5 years OS: 91%

SPIRIT [Preudhomme et al. 2010]	CML patients in CP de novo	600mg/d	At 12 months 65%	At 12/24 months 49%/53%	At 2 years PFS: 96,9%/OS NR
		400 mg/d + IFN alpha	66%	57%/64%	At 2 years PFS: 96,8%/OS NR

Nordic trial [Simonsson et al. 2011]	CML patients in CP de novo	400 mg/d	At 52 weeks 83,9%	At 12 months 54%	NR
		400 mg/d +PegIFN	91,1%	82%	NR

MDACC study [Cortes et al. 2011]	CML patients in CP de novo	800 mg/d	At 12 months 87%	At 12 months 77%	NR
		800 mg/d +PegIFN	90%	77%	NR

NR – not reported
